# UPLC-MS/MS analysis and biological activity of the potato cyst nematode hatching stimulant, solanoeclepin A, in the root exudate of *Solanum* spp.

**DOI:** 10.1007/s00425-021-03766-2

**Published:** 2021-11-02

**Authors:** Alessandra Guerrieri, Kristýna Floková, Lieke E. Vlaar, Mario L. Schilder, Gertjan Kramer, Aleksandra Chojnacka, Yannick R. van Dijk, Harro J. Bouwmeester, Lemeng Dong

**Affiliations:** 1 Plant Hormone Biology Group, Swammerdam Institute for Life Sciences (SILS), University of Amsterdam, Amsterdam, The Netherlands; 2 Mass Spectrometry of Biomolecules, Swammerdam Institute for Life Sciences (SILS), University of Amsterdam, Amsterdam, The Netherlands; 3 Biomolecular System Analytics, Van’t Hoff Institute for Molecular Sciences (HIMS), University of Amsterdam, Amsterdam, The Netherlands

**Keywords:** Analytical chemistry, Eclepins, Hatching assay, Solid phase extraction (SPE), Tomato root exudates

## Abstract

**Main conclusion:**

Solanoeclepin A is a hatching stimulant for potato cyst nematode in very low (pM) concentrations. We report a highly sensitive method for the analysis of SolA in plant root exudates using UHPLC-MS/MS and show that there is considerable natural variation in SolA production in *Solanum* spp. corresponding with their hatching inducing activity.

**Abstract:**

Potato cyst nematode (PCN) is a plant root sedentary endoparasite, specialized in the infection of solanaceous species such as potato (*Solanum tuberosum*) and tomato (*Solanum lycopersicum*). Earlier reports (Mulder et al. in Hatching agent for the potato cyst nematode, Patent application No. PCT/NL92/00126, 1996; Schenk et al. in Croat Chem Acta 72:593–606, 1999) showed that solanoeclepin A (SolA), a triterpenoid metabolite that was isolated from the root exudate of potato, induces the hatching of PCN. Its low concentration in potato root exudate has hindered progress in fully understanding its hatching inducing activity and exploitation in the control of PCN. To further investigate the role of SolA in hatching of PCN, the establishment of a highly sensitive analytical method is a prerequisite. Here we present the efficient single-step extraction and UHPLC-MS/MS based analysis for rapid determination of SolA in sub-nanomolar concentrations in tomato root exudate. This method was used to analyze SolA production in different tomato cultivars and related solanaceous species, including the trap crop *Solanum sisymbriifolium*. Hatching assays with PCN, *Globodera pallida*, with root exudates of tomato genotypes revealed a significant positive correlation between SolA concentration and hatching activity. Our results demonstrate that there is natural variation in SolA production within solanaceous species and that this has an effect on PCN hatching. The analytical method we have developed can potentially be used to support breeding for crop genotypes that induce less hatching and may therefore display reduced infection by PCN.

**Supplementary Information:**

The online version contains supplementary material available at 10.1007/s00425-021-03766-2.

## Introduction

Potato cyst nematodes (PCNs), *Globodera pallida* and *Globodera rostochiensis*, are plant parasitic nematodes and form one of the most damaging pests for solanaceous species, including economically valuable crops such as potato, tomato and eggplant (Perry et al. 2018). These nematodes are called ‘cyst nematode’ because they form a cyst, the hardened dead body of the female, produced at the end of the life cycle that remains in the soil after the crop is harvested (Perry et al. 2018). The cyst contains hundreds of eggs that are protected against biotic and abiotic stresses for up to 20 years. When a suitable host is nearby, the eggs hatch in response to hatching stimulants produced by the host roots, after which the hatched juveniles penetrate the root and induce a feeding site called syncytium (Bohlmann 2015; Perry et al. 2018).

**Fig. 1:**
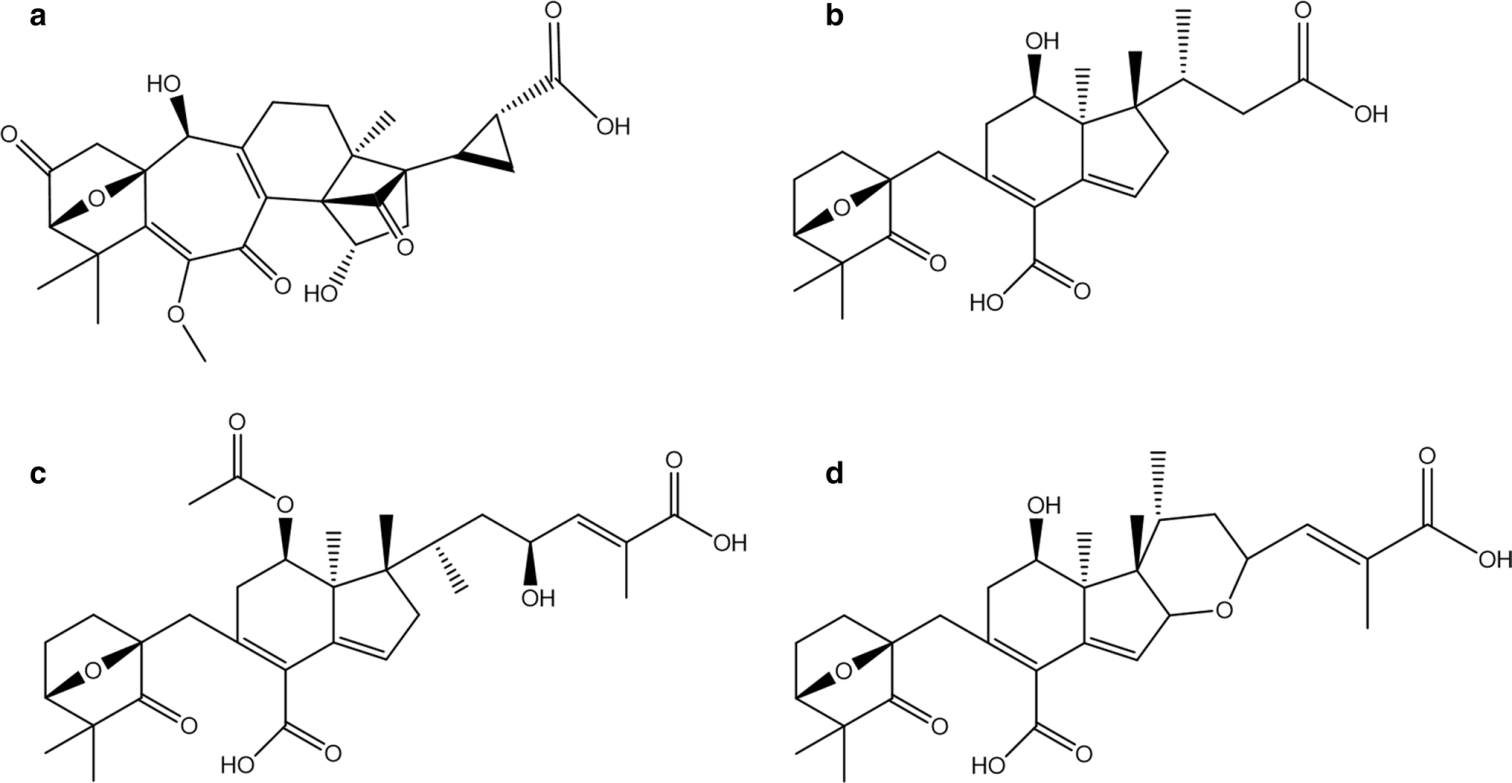
Structures of known hatching factors for cyst nematodes. **a** Solanoeclepin A. **b** Glycinoeclepin A. **c** Glycinoeclepin B. **d** Glycinoeclepin C

Since 1922 a great number of studies have been carried out on identifying hatching factors in potato (Calam et al. 1949a; Massey and Neal 1953; Janzen and Van der Tuin 1956; Devine et al. 1996). The most active hatching stimulant so far isolated from potato root exudate was coined solanoeclepin A by Mulder et al. (1996) (Fig. 1). The structure of this compound was further described in 1999 by Schenk et al. (1999). SolA is a triterpenoid acid with a similar structure as the hatching stimulants of soybean cyst nematode, reported in kidney bean, glycinoeclepin A, B and C (Fig. 1) (Masamune et al. 1982; Fukuzawa et al. 1985).

According to Mulder et al. (1996) SolA was first isolated from the root exudate of 700 potato plants grown in hydroponics. The nutrient solution on which the plants were grown was passed through a column containing Amberlite resin to adsorb the hatching agents, which were subsequently eluted from the column and subjected to a series of purification and fractionation steps. The fractions obtained were tested with hatching assays to determine which fractions induced hatching. The fractions that showed hatching activity were finally combined and concentrated to obtain 245 µg of pure hatching agent whose structure was then determined (Mulder et al. 1996). Tanino et al. (2011) reported for the first time the complete chemical synthesis of SolA. The activity of the synthetic SolA was tested in a series of hatching assays with *G. rostochiensis*, and its hatching activity at concentrations as low as 1 × 10^–8^–1 × 10^–10^ g ml^−1^ water was confirmed. Moreover the hatching activity was compared to the activity of a tomato root exudate concentrated from 500 l nutrient solution used to grow 12,000 tomato plants in hydroponics (Tanino et al. 2011). The activity of the synthetic SolA was only 65% of the activity of the tomato root exudate, suggesting the presence of other hatching factors (Tanino et al. 2011). Shimizu et al. (2020) tested the hatching activity of the major steroidal glycoalkaloids in potato and tomato root exudates, on *G. rostochiensis.* Despite the significant hatching stimulant activity of all the tested compounds, including α-solanine, α-chaconine and α-tomatine, however, when compared to SolA the activities were quite weak.

The direct application of ‘hatching factors’ into the soil to control the spreading of PCN has been proposed, the so-called ‘suicide hatch’ strategy. For example, Devine and Jones (2000) demonstrated that the exogenous application of hatching factors in the field before planting, resulted in a 50% reduction in the population of *G. rostochiensis*, due to the high mortality of the juveniles in the absence of their plant host. The ‘hatching factors’ were obtained by concentrating tomato and potato root exudates from 7000 and 40 plants grown in hydroponic culture and pots, respectively, using Amberlite resin. A similar approach is the trap-crop strategy, employing a crop that induces hatching of the cyst nematodes but is not a suitable host or is destroyed before the host has completed its life cycle, thus reducing the infestation level (Scholte 2000a). *Solanum nigrum* and *Solanum sisymbriifolium* have been proposed as potential trap crops to control PCN (Scholte 2000b). In both the suicide hatch and trap crop approach, it is unknown whether SolA is responsible for the hatching activity, or whether other factors induce the hatching response in potato cyst nematodes. Both Mulder et al. (1996) and Schenk et al. (1999) did not provide insight into the concentration of SolA in potato or tomato root exudates. Thus, it is difficult to provide advice on the precise amount of root exudates to apply to reach the desired effect of PCN control. Likewise, without knowledge of the hatching factor identity and concentration in the root exudate of trap crops it is difficult to optimize this PCN control strategy.

Up to now, one of the indirect methods to quantify SolA is the hatching assay performed on cysts or eggs of PCN (Been and Schomaker 2001). However, this method is not very accurate, since large variation can be introduced by using different nematode species/pathotypes and root exudates (Devine and Jones 2001a; b). As mentioned before, hatching assays can be influenced by the presence of different hatching factors in the root exudates as recently confirmed (Ochola et al. 2020). It has already been reported by Byrne et al. (1998), that hatching factors such as α-solanine and α-chaconine induce a dosage-dependent response in *G. rostochiensis*. Moreover, at concentrations above 1 × 10^–4^ M, these compounds inhibited hatching (Byrne et al. 1998). Finally, hatching assays lack a standard procedure and require the use of potato cyst nematodes which are quarantine organisms in many countries.

Strikingly, even though there are several indications, there is no direct evidence for the presence of SolA in tomato root exudate. The presence of SolA has so far only been demonstrated in potato root exudates as described in the patent from Mulder et al. (1996)*.* In the patent the method is claimed to be usable also for the isolation of SolA from tomato root exudates. Schenk et al. (1999) reported that their procedure could be used to isolate a compound from tomato root exudate with the same structure as SolA, but it is unclear from their paper which genotype of tomato was used, how it was grown and how many plants were used. A better insight into the presence of SolA in the root exudate of crop genotypes and its role in PCN hatching can open up the possibility for breeding new genotypes of plants with low hatching activity, *i.e.* a low concentration of hatching factors, including SolA.

Thus, there is a great need to quantify the SolA production in root exudates and further discover the importance of this hatching factor. The techniques so far described in the literature are laborious, including adsorption on active charcoal, ether extraction of acidified aqueous samples and fractionation, and silica gel column chromatography (Calam et al. 1949b; Marrian et al. 1949) and therefore not suitable for routine analysis. Moreover, there is a lack of information on compound loss and recovery. Also, large amounts of plants and root exudate are needed to isolate sufficient SolA.

Therefore, we set out to establish an UHPLC-MS/MS-based method for the fast and sensitive analysis of the SolA concentration in root exudates. Hereto, we used a single-step purification step, employing a mixed-mode anion exchange sorbent, which efficiently pre-concentrates acidic analytes and improves sample purity. With this method, we can quantify SolA in 5 ml of root exudate of a single plant. Using our new approach, we detected and quantified SolA in root exudates of cultivated tomato (*Solanum lycopersicum* cv Moneymaker and cv MicroTom), their wild relatives (*Solanum pennellii*, *Solanum pimpinellifolium, Solanum habrochaites*) and the commonly used trap-crop *S. sisymbriifolium*. By comparing the SolA concentration in root exudates of different solanaceous species with the hatching response of the PCN *G. pallida* we show evidence of the importance of SolA in determining the hatching activity of these species.

## Materials and methods

### Chemicals and materials

An authentic standard of solanoeclepin A was kindly provided by Prof. Keiji Tanino (Hokkaido University, Japan). The methanol, acetonitrile, deionized water and formic acid for UPLC-MS/MS and LC-ESI-QTOF-MS analysis were all hypergrade for LC–MS, purchased from Biosolve BV (Valkenswaard, The Netherlands). The KOH and ammonia for sample extraction and purification were obtained from Merck (Darmstadt, Germany). Deionized (Milli-Q) water was prepared using a water purification system Milli-Q^®^ (Merck Millipore, Burlington, MA, USA).

### Plant growing and sample collection

Seeds of tomato (*S. lycopersicum* cv Moneymaker and MicroTom) were obtained from our greenhouse staff, seeds of the wild tomato species (*S. pimpinellifolium, S. pennellii* and *S. habrochaites* accessions LA1777 and PI127826) were obtained from Enza Zaden (Enkhuizen, The Netherlands); seeds from *S. sisymbriifolium* cv Pion and Quattro, were obtained from Vandinter Research b.v. (Scheemda, The Netherlands).

All seeds were germinated in potting soil in a greenhouse at 24 °C, 60% relative humidity with a 16 h photoperiod and watered with rainwater. After germination and cotyledon emergence, seedlings were transferred to pots (15 cm diameter and 13 cm height) and grown under the same conditions as for the germination.

Preliminary experiments with tomato showed that SolA can be detected in the root exudate after 2 weeks. It’s amount increases until 3 weeks after which it stabilizes until 5 weeks. Thus, 4 weeks old plants were chosen as a standard time point to measure SolA, also because then all the species were in the same phenological stage.

Root exudate collection from five biological replicates was performed on 4-week-old plants: distilled water was poured onto the soil and the flow through from the pot was collected in a beaker until the amount of 300 ml was reached. This amount was previously evaluated to be sufficient to accurately represent the root exudate composition.

Roots of the plants were also collected by washing them and carefully removing the soil particles. The roots were then dried on paper and the fresh weight was recorded (Suppl. Table S2).

Of the 300 ml collected exudate, 50 ml were stored at 4 °C for further analysis for all the genotypes. For the method development, the remaining 250 ml root exudate of all biological replicates of *S. lycopersicum* cv Moneymaker were pooled and stored for further use.

### The stability of solanoeclepin A

The stability in tested solutions 20%, 50% and 100% methanol/water (v/v) was monitored over 4 weeks. Solutions containing known amount of SolA (5 pmol ml^−1^) were kept at − 20 °C. Before each measurement, the 0.5 ml of solution (2.5 pmol) was transferred to a new vial and evaporated to dryness using ScanVac vacuum concentrator ScanSpeed 40 (LaboGene, Allerød, Denmark). Samples were reconstituted in 100 µl of 20% methanol (v/v) and analyzed by UPLC-MS/MS. The instrument response was normalized by the signal of freshly prepared standard solutions (in duplicates) before each analysis. Following measurements of these solutions contributed to the set of 1, 2, 3 or 4-week-old samples. Results were calculated as the percentage of mean peak area measured in the set of 1, 2, 3 or 4-week-old samples in octuplicates, compared to the average of standard area, freshly prepared each week.

The short-term stability of SolA was tested in 100% methanol, 0.2 M formic acid in methanol and tomato root exudates (TRE, *S. lycopersicum* cv Moneymaker, prepared as mentioned above). 0.5 ml of these solutions was spiked with 2.5 pmol of SolA and samples were kept for 2 h at 4 °C. Samples were evaporated in vacuo, reconstituted and analyzed by UHPLC-MS/MS for compound recovery analysis. Additionally, in case of TRE, dried sample matrix was spiked with 2.5 pmol SolA to correct for the effect of the matrix on the signal of the analyte.

### Sample extraction and purification

For sample processing, the root exudates were filtered over filter paper to remove soil particles and the pH was measured and corrected to around 6.9–7.2 using a 1 M solution of KOH. Five ml of root exudate was applied to an SPE Oasis^®^ MAX (3 cc/60 mg, Waters, Milford, MA, USA) column purchased from Waters Co., pre-conditioned with one column volume (3 ml) of 100% methanol and activated with one column volume of 5% NH_4_OH/H_2_O (v/v).

The retained sample was washed with one column volume of deionized (Milli-Q) water to remove salts and a following wash with one volume of 100% methanol was applied to elute low-polar contaminants, retained by reversed phase only. SolA elution from the cartridge was achieved with one column volume 0.2 M formic acid in methanol which was collected in a 4 ml glass vial. Samples were evaporated until dryness in vacuo and reconstituted with 100 µl of 20% methanol in water (v/v) for UHPLC-MS/MS analysis.

### UHPLC-MS/MS analysis

The analysis of SolA was performed using a Waters Acquity ultra-high pressure liquid chromatography (UHPLC)™ I-Class System (Waters) equipped with a binary solvent manager and sample manager, coupled to a Xevo^®^ TQ-XS tandem quadrupole mass spectrometer (MS/MS, Waters MS Technologies, Manchester, UK) with electrospray (ESI) ionization interface. The reconstituted sample was filtered using a micro-spin nylon filter (0.2 μm pore size, Thermo Fisher, Waltham, MA, USA). Subsequently, 5 µl was injected onto the reversed-phase UHPLC column (Acquity UPLC^®^ Ethylene Bridged Hybrid (BEH) C18 column, 2.1 × 100 mm, 1.7 μm particle size, Waters), kept at a constant temperature of 40 °C. For the comparison, another UPLC C18 column (Acquity UPLC^®^ Charged Surface Hybrid (CSH) 2.1 × 100 mm, 1.7 μm particle size, Waters) was also used. The analyte was eluted at a flow rate of 0.3 ml min^−1^ with a 9 min linear gradient of 15 mM formic acid/water (A) and 15 mM formic acid/acetonitrile (B) with the following elution profile: 0–1 min (5% B), 6 min (50% B), 8 min (80% B), 9 min (95% B). At the end of the gradient, the column was washed with 95% B for 1 min and finally equilibrated to initial conditions for 2 min. The eluent was introduced into the ESI ion source of the mass spectrometer, operating at optimized settings: capillary/cone voltage (1200/ 30 V), source temperature (120 °C), desolvation temperature (550 °C) and drying gas flow (1000 l h^−1^). SolA was analyzed in positive mode as [M + H]^+^, using diagnostic and confirming precursor-to-product transitions 499 > 83, > 399, > 315, > 453 with optimized collision energy 30, 25, 25, and 20 eV, respectively, and collision gas (argon) flow of 0.15 ml min^−1^. The instrument control, MS data acquisition, and processing were carried out by the MassLynx™ software, version 4.2 (Waters).

### LC-ESI-QTOF-MS analysis

SolA (10 μM) was analyzed using a QTOF MS equipped with a dual-stage trapped ion mobility separation cell (timsTOF pro Bruker Daltonics Inc, Billerica, MA, USA). Sample injection (40 μl) and LC separation were performed on an Ultimate RS UHPLC system (Thermo Scientific, Germeringen, Germany) with an Acquity UPLC CSH C18 130 Å, 1.7 µm, 2.1 mm × 100 mm protected by a VanGuard 2.1 mm × 5 mm of the same material. A gradient from 1% acetonitrile to 99% acetonitrile in 18 min was applied at 0.4 ml min^−1^ (solvent A 0.1% formic acid in water, solvent B 0.1% formic acid in acetonitrile), before returning to initial conditions. Eluting compounds were sprayed in positive ion mode by an Apollo II ion funnel ESI source (Bruker Daltonics Inc). Source settings were: capillary voltage 4500 V; end plate offset 500 V; drying temperature 220 °C; desolvation gas (nitrogen) flow 8.0 l min^−1^; nebulizer gas pressure 2.2 bar. Samples were analyzed in timsoff mode, auto MS/MS settings were: switching threshold 500 cts; cycle time 0.5 s; active exclusion after 3 spectra; release after 0.2 min. Analytes selected for fragmentation were fragmented by collision with nitrogen gas at a collision energy of 30 eV. Precursors and fragments were analyzed by the time of flight analyzer using a range of 100–1350 *m/z*. Resulting data were analyzed using DataAnalysis ver. 4.3 (Bruker Daltonics).

### Fractionation of root exudates

To determine whether hatching activity and SolA co-eluted, we fractioned a tomato root exudate on UHPLC. Hereto, 1 ml of root exudate from Moneymaker was freeze dried. Salts were precipitated by dissolving the dried exudate in 1 ml of methanol. The sample was centrifuged at 3000*g* for 3 min, and the supernatant was transferred to a clean vial prior to drying by vacuum evaporation. The sample was then re-dissolved in 150 µl 25% ACN and injected on UHPLC. Separation of the samples was achieved using the same gradient and same column as used for the analysis. The eluent was fractionated using a Waters fraction manager. The sample was injected 8 times, and the SolA fraction, spanning 30 s of the chromatogram, was collected from each run and pooled. The fraction was again freeze dried, dissolved in 0.4 ml 2% ethanol and tested for hatching activity.

### Globodera pallida hatching assay

*G. pallida* D383 pathotype Pa3 was reared in a greenhouse on potatoes (*Solanum tuberosum*) and subsequently stored at 4 °C, which broke the diapause, until use for all hatching assays. About 50 first generation cysts were soaked in tap water for 1 week at 20 °C in the dark. Then, the cysts were carefully opened and the eggs were collected in tap water. The egg suspension was distributed in aliquots of 100 µL into the wells of a glass-coated 96-wells plate with 50–100 eggs per well. Subsequently, 100 µl tenfold diluted unpurified root exudate, SolA standard of different concentrations in 2% f in tap water, or tap water was added to each well. All conditions were tested in triplicate and concentrations given in the Results section are calculated according to the end concentration in the 200 µl in each well. Photos were taken of individual well at the start of the assay, and again after plates were incubated for 7 and 11 days at 20 °C in the dark. Eggs and hatched juveniles were counted twice on each photo and hatching percentage was calculated according to:

$$\left( {J_{t11} - J_{t0} } \right)/J_{t0} \times 100,$$

where *J*_*t*11_ is the number of hatched *J*_2_ after 11 days of treatment, *J*_*t*0_ is the number of hatched *J*_2_ at the start of the assay, and *E*_*t*0_ is the number of eggs at the start of the assay.

### Statistical analysis

Comparison of SolA levels in different solanaceous species was carried using a one-way ANOVA analysis in R. Homogeneity of variation and normal distribution of hatching data were verified with Levene’s test and the Shapiro–Wilk test, respectively. Multiple pairwise comparison Tukey test in *R* was used to determine differences in hatching percentages between root exudates of different solanaceous species.

## Results

### UHPLC-MS/MS method development

To analyse SolA levels in complex real samples with sufficient sensitivity, a selective extraction procedure was developed and combined with optimized UHPLC-MS/MS analysis. For chromatography two different reversed-phase-based UPLC C18 columns (CSH and BEH) were compared. These polymer-based UPLC columns were successfully used in the analysis of multiple plant hormones (Floková et al. 2020). The additional charge on the surface of the CSH particles would theoretically provide a better peak shape for acidic compounds (Urbanová et al. 2013; Floková et al. 2014). However, in a linear 9 min gradient with 15 mM formic acid in the mobile phase, SolA was detected as a late-eluting broadened asymmetrical peak using the CSH column (Suppl. Fig. S1). Under the same conditions, a 1.5 min shorter retention time and excellent peak shape of the analyte were achieved using the BEH column, which was therefore employed in all further experiments. Using further optimized conditions (see “Materials and methods”), a stable and reproducible retention time was achieved with coefficient of variation 0.43% (*n* = 191).

**Fig. 2:**
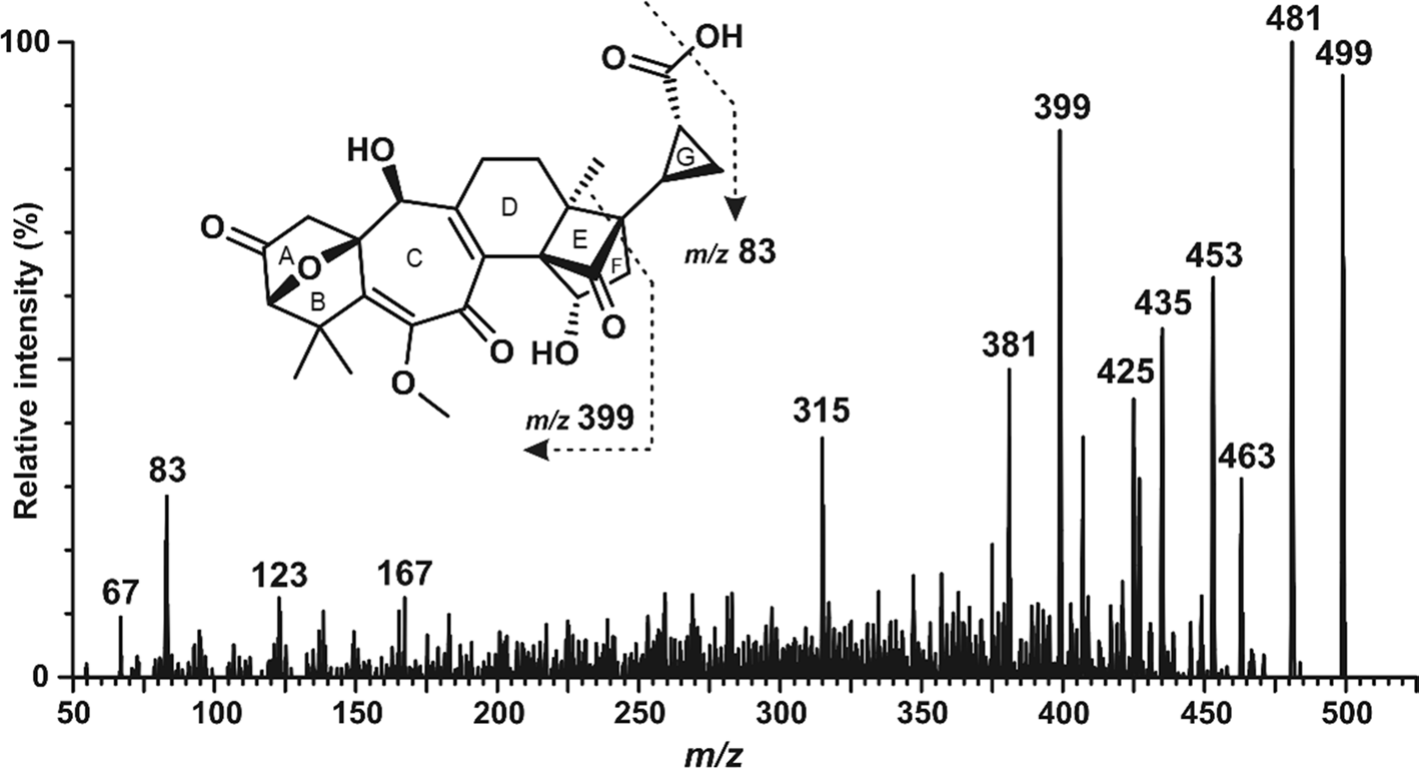
Product-ion spectra of SolA and predicted fragmentation pattern

Injection of SolA into the UHPLC-MS/MS provides both deprotonated [M-H]^−^ and protonated [M + H]^+^ molecular ions of *m/z* 497 and 499, respectively, with a stronger signal in the positive ESI mode (Suppl. Fig. S2). The fragmentation pattern of the positively charged molecular ion was further investigated and appropriate precursor-to-product ion transitions for multiple-reaction monitoring (MRM) analysis were selected. The diagnostic product ion (*m/z* 399; molecular formula C_22_H_23_O_7_) is characterized by the loss of the SolA cyclopropane carboxylic acid side chain (G-ring) and a methyl group. The fragment (*m/z* 83) corresponds to the dehydrated cation of the side chain (G-ring) including a methyl, derived from the E/F ring (C_5_H_7_O) (Suppl. Fig. S3b). Other detected product ions in the daughter spectra represent the rearranged ABCD ring system (*m/z* 315; molecular formula C_19_H_23_O_4_) (Suppl. Fig. S3b), the loss of the carboxy group (*m/z* 453; molecular formula C_26_H_29_O_7_) and the loss of water (*m/z* 481; molecular formula C_27_H_29_O_8_) (Fig. 2). The collision energy for each of the selected MRM transitions 499 > 399, > 83, > 315, > 453 was optimized and all MS/MS conditions, including settings of the mass spectrometer, such as capillary voltage, source/desolvation temperature, desolvation/collision gas flow and cone voltage as listed in Materials and methods. The accurate mass of these fragments was obtained by liquid chromatography followed by high resolution–mass spectrometry (Suppl. Fig. S4).

### Solanoeclepin A extraction and purification procedure

Sample pre-concentration and clean-up based on solid-phase extraction (SPE) is the strategy to simplify complex sample matrices before compound analysis by UHPLC-MS/MS. SolA with its rearranged tetranortriterpene structure (Fig. 1a) is a polar molecule in which the methyl enol ether and cyclobutanone moieties could be susceptible to hydrolysis in basic and strong acidic conditions (Tanino et al. 2011).

To design a suitable extraction and purification procedure, we tested the chemical stability of SolA. Considering the polarity of SolA and the sample origin (root exudates), the water-miscible methanol was initially selected as the solubilizing solvent for further sample processing. The stability of SolA was monitored in 100% methanol and its 50% and 20% aqueous solutions (v/v) over 4 weeks. The percentage of methanol was chosen to achieve efficient solubility of the compound for LC–MS, long-term sample stability and to meet compatibility with conditions of reversed-phase based SPE sorbents. Samples were dried in vacuo and compound recovery was analyzed by UHPLC-MS/MS. Due to the absence of an appropriate internal standard, inter-week differences of the instrument response were normalized by weekly analysis of freshly prepared standard solutions (2.5 pmol/sample) in duplicates. The average compound recovery was 87% with no significant differences between individual sets of measurements as well as solutions (Suppl. Fig. S5).

Subsequently, the short-term stability of SolA in root exudates and acidified methanol was analyzed to evaluate critical sample collection and SPE elution conditions. Triplicates of tomato root exudates (TRE) and 0.2 M formic acid in methanol were spiked with 2.5 pmol of SolA and kept at 4 °C for 2 h. Samples were dried in vacuo and analyzed for compound recovery. The response of SolA, spiked in the dry sample matrix, was analyzed to allow for compensation of the matrix effect on ionization efficiency. Spiked SolA loss in the background of tomato root exudate was 14.1%. The presence of 0.2 M formic acid in methanol slightly decreased SolA recovery by 8% compared to pure methanol (Suppl. Table S1).

The presence of the carboxylic group in the structure of SolA provides an opportunity for selective analyte purification through anion exchange chromatography. Therefore, the polymer-based SPE Oasis^®^ MAX (Waters), combining reversed phase with anion exchange retention mechanisms, were used for SolA isolation. Tomato root exudates of which the pH was adjusted to around 6.9–7.2, were applied on the sorbent that was pre-conditioned with methanol and activated with 5% aqueous ammonia. The column was then washed with one column volume of water to remove salts. The following wash with 100% methanol was applied to elute low-polar contaminants, retained by reversed phase only. SolA elution was achieved with 0.2 M formic acid in methanol (Suppl. Fig. S6).

To evaluate the extraction recovery of this sample preparation procedure, quadruplicates of TRE (5 ml) were spiked with 2.5 pmol of SolA and further purified using the Oasis® MAX cartridge (Waters). Samples were evaporated to dryness, reconstituted in 100 µl of 20% methanol/water (v/v) and analyzed by UHPLC-MS/MS. Since an internal standard was not available the effect of the matrix on instrument ionization efficiency was analyzed by comparing the instrument response with the SolA standard, spiked in the dry purified sample matrix. The SolA in the presence of sample matrix was recovered after SPE from 90.3% (Suppl. Table S1). For comparison, we examined the SolA extraction recovery also from water and obtained only 6.1% loss. These data suggest a slight decrease in the extraction efficiency in the presence of sample contaminants. Based on these results we concluded that the purification protocol using Oasis^®^ MAX sorbent is suitable for SolA extraction from tomato root exudates. The hatching activity of partially purified (with Oasis^®^ MAX cartridge) root exudate of *S. lycopersicum* cv. Moneymaker was evaluated and was not different from the crude root exudate (Suppl. Fig. S7).

### Method validation

To determine the sensitivity parameters of the newly developed UHPLC-MS/MS method, a 13-point calibration curve was constructed based on the response areas of the serially diluted authentic standard, injected in quadruplicate. The amount of analyte ranged between 240 amol and 1 pmol (Suppl. Fig. S8a). The instrument detection limit was defined as three times signal-to-noise ratio (S/N) and this corresponds to 1 fmol of injected SolA. The response was linear over four orders of magnitude with a correlation coefficient of 0.998 and the limit of quantitation 0.002 pmol per injection (Suppl. Fig. S8a, b).

The effectiveness of the method was further evaluated by standard addition using tomato root exudates. Samples (5 ml, quadruplicate) were spiked with the authentic standard of SolA at two concentration levels (1 and 2.5 pmol) prior to SPE purification. Purified samples were analyzed by UHPLC-MS/MS and SolA concentrations were calculated individually using two sets of 8-point calibration curves (7.8 fmol–1 pmol), prepared in blank solvent (external calibration) and purified sample matrix (matrix-matched calibration). Non-spiked extracts of tomato root exudates were analyzed to subtract endogenous SolA levels from the amount of added standard. The overall analytical precision for both spiked concentration levels calculated from external calibration was determined as 4.65% relative standard deviation (RSD) (Table 1). The method accuracy was assessed as 92.5% and 79% of the correct 1 and 2.5 pmol concentration levels, respectively. Both, method precision and accuracy, were improved using matrix-matched calibration that compensates for the ion suppression in the presence of sample matrix (Table 1).

**Table 1:** Method validation

Tomato root exudates	Spiked amount (pmol)	Determined content (pmol) ± Standard deviation	Precision (% RSD)	Accuracy (% bias)
External calibration	1	0.93 ± 0.04	4.72	− 7.44
	2.5	1.97 ± 0.09	4.59	− 21.04
Matrix-matched calibration	1	0.98 ± 0.01	0.99	− 2.30
	2.5	2.11 ± 0.05	2.21	− 15.47
Extraction solvent				
External calibration	1	1.08 ± 0.08	7.71	7.81
	2.5	2.37 ± 0.11	4.84	− 5.19

The precision and accuracy of SPE sample preparation was calculated using an 8-point calibration curve, prepared in 20% methanol/water, v/v (external calibration) and purified samples of tomato root exudates (reconstituted in 20% methanol/water, v/v; matrix-matched calibration). Tomato root exudates (5 ml) and control samples of extraction solvent (5 ml of water) were spiked with 1 and 2.5 pmol of SolA standard. Samples were purified using Oasis® MAX SPE sorbent and analyzed by UHPLC-MS/MS. All experiments were performed in quadruplicates. The method’s precision is determining relative standard deviation (% RSD) for each spiked level. The accuracy is assessed as difference of determined content from spiked content in percentage (% bias)

### Solanoeclepin A is produced by several different solanaceous species

**Fig. 3:**
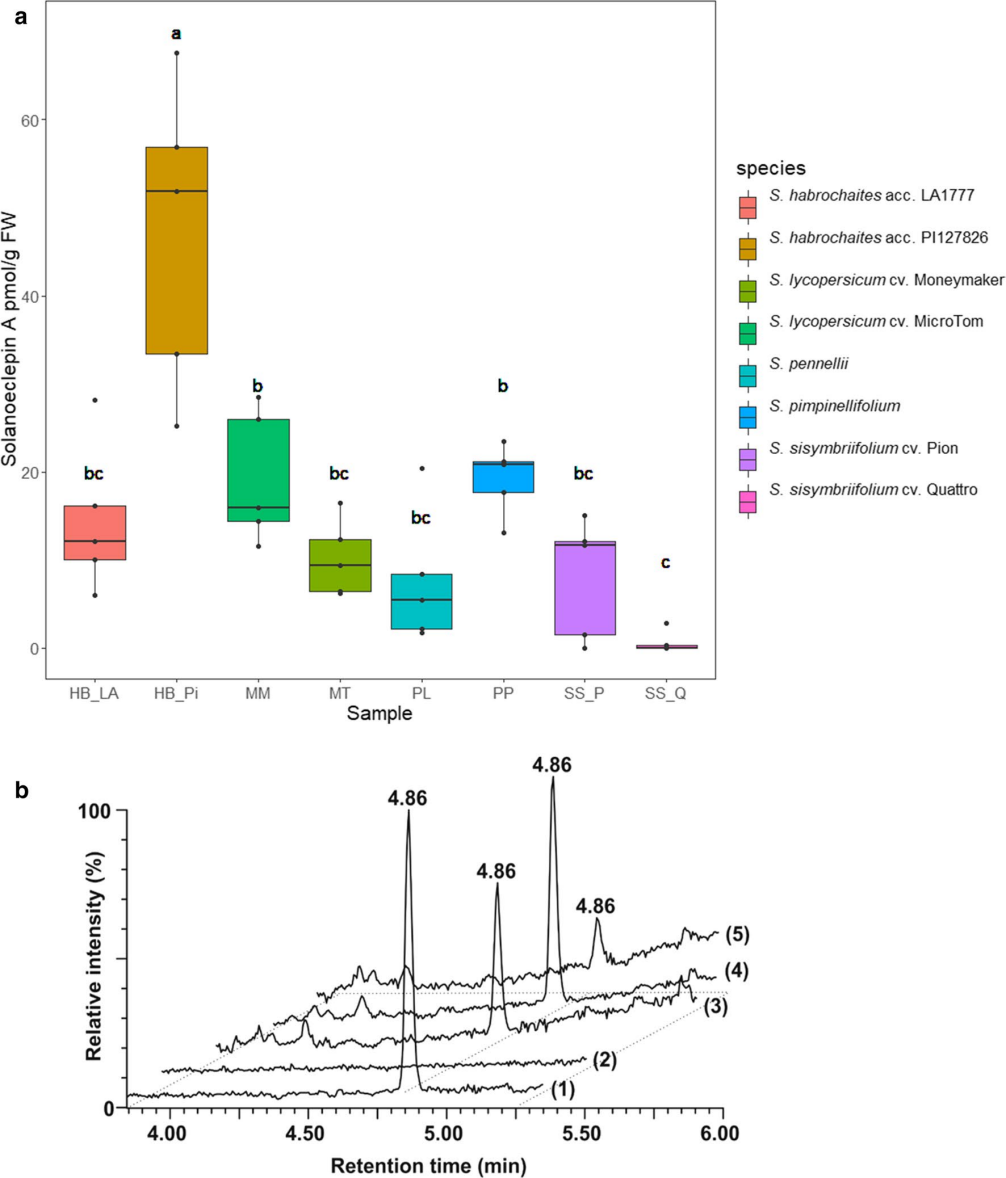
**a** SolA concentration in root exudates of different Solanaceae species, *S. habrochaites* acc. LA177 (HB_LA), *S. habrochaites* acc. PI127826 (HB_Pi), *S. pennellii* (PL) and *S. pimpinellifolium* (PP), tomato cultivars: *S. lycopersicum* cv. Moneymaker (MM) and MicroTom (MT) and trap crop: *Solanum sisymbriifolium* cv. Pion (SS_P) and cv. Quattro (SS_Q). The concentration is expressed in pmol/gram of fresh root weight (pmol g^−1^ FW). Black dots represent SolA concentration from each biological replicate. Samples were analyzed in five replicates, median (line within the box), first and third quartiles (box), non-outlier range (whiskers), and outliers (dot) are shown. Different letters indicate statistically significant differences between samples using a one-way ANOVA analysis. **b** LC–MS chromatograms of SolA standard (1), negative control (2) and real samples of root exudates obtained from *S. lycopersicum* cv. Moneymaker (3), *S. habrochaites* acc. PI127826 (4) and *S. sisymbriifolium* cv. Quattro (5)

Having established an effective SPE protocol for SolA extraction, we quantified the SolA concentrations in root exudates of cultivated tomato (*S. lycopersicum* cv. Moneymaker), model tomato genotype MicroTom, three wild tomato relatives (*S. pimpinellifolium, S. pennellii* and *S. habrochaites* accessions LA1777 and PI127826) and two cultivars of the trap crop *S. sisymbriifolium* cv. Pion and Quattro. SolA was detected in all these genotypes (Fig. 3, Suppl. Fig. S8c). There was a significant difference in the SolA production by the two accessions of *S. habrochaites*, with the highest level of SolA produced by PI127826 (67.6 pmol g^−1^ FW) (Fig. 3 and Suppl. Table. S2). *S. sisymbriifolium* cv. Quattro produced the lowest amount of SolA with a concentration below 2.83 pmol g^−1^ FW. On the other hand, no significant difference in SolA production of *S. sisymbriifolium* cv. Pion, *S. pennelli* and *S. lycopersicum* cv MicroTom. Surprisingly, both cultivars of the trap crop produced less SolA than the commercial tomato cultivar Moneymaker, of which the production is comparable to the wild *S. pimpinellifolium* (11.6 – 28.4 pmol g^−1^ FW). The complete dataset is shown in the Supplemental Table S2.

### Hatching of *G. pallida* eggs under influence of root exudates of solanaceous species

**Fig. 4:**
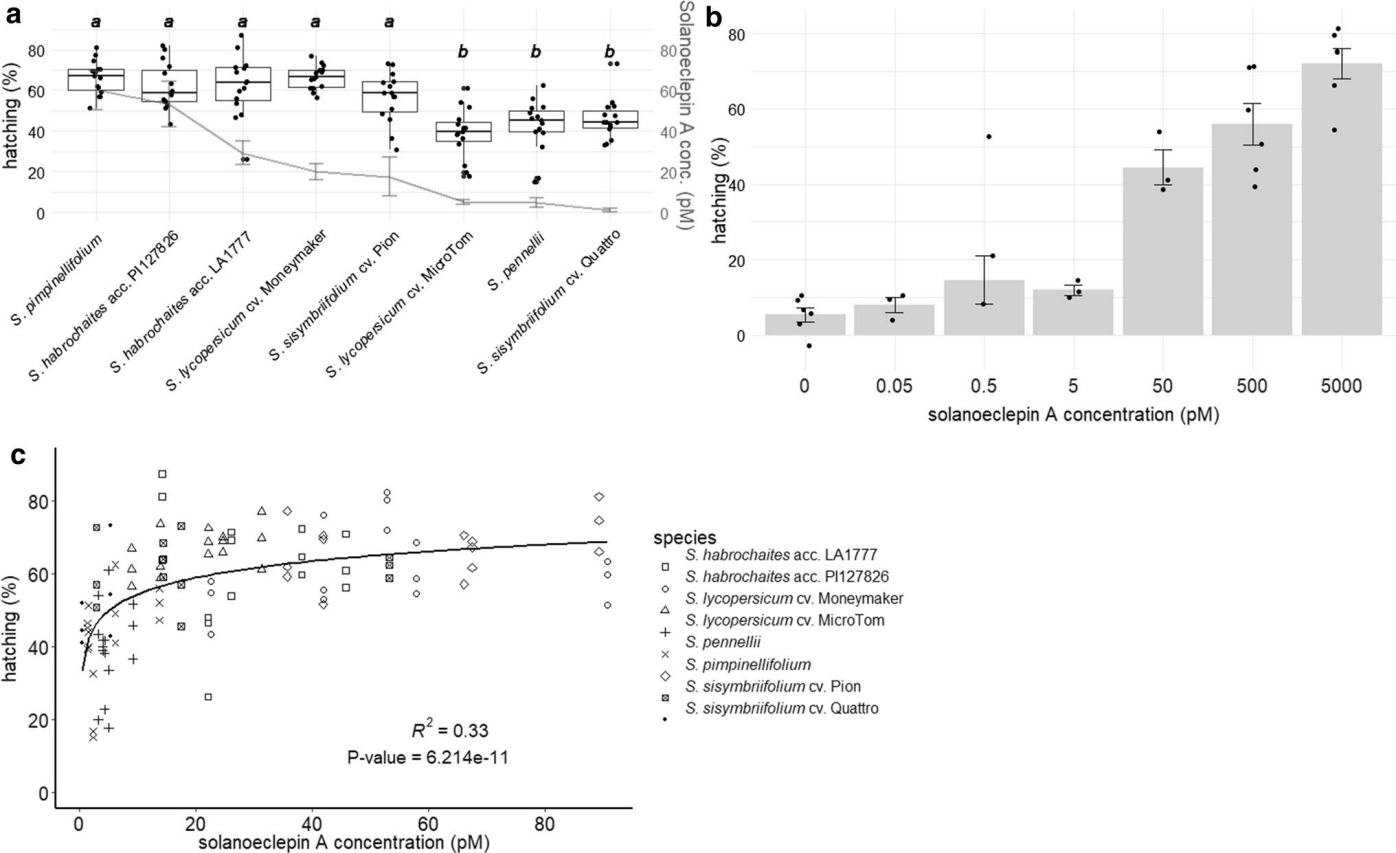
**a** Boxplots of hatching of *G. pallida* eggs treated with root exudates of solanaceous species. Significant differences (*P* < 0.05) are indicated with a and b. The line graph shows the SolA concentrations in the root exudates with error bars (standard error, *n* = 5). **b** Hatching percentages of *G. pallida* eggs treated with standard solutions of SolA. Bars indicate the mean hatching induced by the respective treatment, error bars indicate standard error, black dots are individual measurements. One outlier at 0.5 pM was ignored in the calculation of the mean and standard error. **c** Mean percentages of hatching of *G. pallida* eggs as induced by root exudates of solanaceous species (*S. pimpinellifolium, S. habrochaites* acc. PI127826 and acc. LA1777, *S. lycopersicum* cv. Money maker and cv. MicroTom, *S. sisymbriifolium* cv. Pion and cv. Quattro, and *S. pennellii*) described in this paper, of which the SolA content was known. Line shows the log relationship between SolA concentration and hatching of *G. pallida* eggs. The *P*-value indicates that the *R*^2^ value of 0.33 is significant

The effect of the root exudates of these solanaceous species on hatching of *G. pallida* eggs was evaluated using a hatching bioassay (Suppl. Fig. S6, Fig. 4a). Crude exudates were diluted tenfold, since previous results showed that this provides an optimum concentration for a reproducible hatching assay (Suppl. Fig. S9). There was a relatively large variation between the replicates of root exudate samples from the same species (Suppl. Fig. S6). However, all root exudate replicates showed higher hatching compared to tap water (Suppl. Fig. S6). SolA standard solutions of 0.5 and 5 nM showed comparable hatching (~ 55% and 75%, respectively) as the most potent root exudates (Suppl. Fig. S6).

There is a significant difference in induced hatching between two groups of exudates: the root exudates of *S. lycopersicum* cv. Moneymaker, *S. pimpinellifolium*, *S. habrochaites* acc. LA1777, *S. habrochaites* acc. PI127826 and *S. sisymbriifolium* cv. Pion induced higher hatching than those of *S. pennellii, S. sisymbriifolium* cv. Quattro and *S. lycopersicum* cv. MicroTom (Fig. 4a). The commercial cultivar *S. lycopersicum* cv. Moneymaker induced the highest mean hatching rate, whereas *S. lycopersicum* cv. MicroTom induced the lowest mean hatching rate. Interestingly, the widely used trap crop *S. sisymbriifolium* cv. Pion induced a mean hatching rate of around 45%, which signifies its suitability as a trap crop (Dandurand and Knudsen 2016).

Hatching rates induced by root exudates seem to depend to some extent on their SolA content although this is difficult to conclude as in genotypes *S. pimpinellifolium*, *S. habrochaites* acc. PI127826 and LA1777 and in *S. lycopersicum* cv. Moneymaker hatching rate is saturated (Fig. 4a–c). Furthermore, other hatching factors seem to be playing a role, since the non-saturated root exudates (*S. sisymbriifolium* cv. Pion, *S. lycopersicum* MicroTom, *S. pennelli* and *S. sisymbriifolium* cv. Quattro) that contain only low SolA concentrations, still induce relatively high hatching. For example, the root exudate of *S. lycopersicum* cv. MicroTom induced around 40% hatching while it contains only about 5 pM SolA, a concentration that, as pure standard, induces no significant hatching compared with water (Fig. 4b). Similar observations hold for root exudates of *S. pennelli* and *S. sisymbriifolium* cv. Quattro, which contain around 5 and 1 pM SolA, respectively (Fig. 4a). Nevertheless, correlation analysis of hatching and SolA concentration in the root exudate yielded a positive, logarithmic correlation (Fig. 4c). Likely, the low *R*^*2*^ can be explained by the fact that hatching with some exudates was saturated and that there may also be other factors (stimulants and/or inhibitors) other than SolA in the root exudates that affect the hatching rate and therefore the correlation between SolA concentration and hatching. To get a more thorough idea of how much hatching is caused solely by SolA from root exudates, the fraction containing SolA was collected from *S. lycopersicum* cv. Moneymaker and tested for hatching activity (Suppl. Fig. S10). This fraction was able to hatch almost as much eggs as crude root exudate and a standard solution of SolA of 0.5 nM. Hence, overall, there is a reliable positive correlation between SolA concentration and hatching of *G. pallida* eggs making SolA concentration a good predictor of hatching (Fig. 4c).

## Discussion

The present study established, for the first time, an efficient and simple single-step extraction method of SolA from root exudates as well as an ultra-sensitive UPLC MS/MS method to quantify SolA content in plant root exudates. Several attempts of purification and characterization of PCN hatching factors from potato and tomato root exudates have already been reported in the literature from the early 1940s. For example, Calam et al. (1949a,b) proposed a method for the preparation of solid concentrates of PCN hatching factor that included the collection of 100 l of root exudates from 4000 to 5000 plants. The hatching factors in the root exudates were subsequently absorbed on charcoal followed by complicated and time-consuming elution and evaporation steps to obtain a brown powder, which contained 60–70% of the active factor present in the root exudates (Calam et al. 1949a,b). Mulder et al. (1996) described a complicated procedure to isolate 245 µg of SolA from the root exudate of 700 potato plants. Subsequently, Schenk et al. (1999) reported that they managed to purify 200 µg of pure SolA from crude potato root exudate, but the experimental procedure by which this result was achieved was not included. Later studies have only reported the method for chemical synthesis of SolA, without investigating the extraction procedure of this compound from plant root exudates (Tanino et al. 2011).

The method developed here was subsequently used on several different solanaceous species, showing that the method is suitable for investigating the natural variation in SolA levels in tomato, but also other solanaceous species such as wild tomato relatives and the trap crop *S. sisymbriifolium*. Interestingly, we found significant differences between different accessions of the same species such as *S. habrochaites* acc. PI127826 and acc. LA1777. This result warrants further investigation as the two accessions come from the same center of origin in Peru. Indeed, it has been shown that different *S. habrochaites* accessions can display large chemical diversity, for example in terpenoid production the glandular trichomes (Gonzales-Vigil et al. 2012). Since the method presented here is relatively easy to use, sensitive and fast, it can be applied in high-throughput screening approaches to select genotypes that produce lower amounts of hatching stimulant and can potentially be used to breed for reduced PCN infection.

Using our new method, we showed that root exudate of the trap crop *S. sisymbriifolium* contains SolA and the hatching assay confirmed its hatching activity for PCN reported before (Scholte 2000b). This finding is different from the results presented by Sasaki-Crawley et al. (2010), who suggested that *S. sisymbriifolium* produces other hatching stimulants, not SolA. This discrepancy is likely caused by the improved analytical method that we have developed and/or by the analysis of different cultivars*.* Indeed, of the two cultivars that we analyzed, only one is producing a clearly detectable amount of SolA. Our new method opens up the possibility to investigate and optimise the efficiency of the cultivars that are currently used as trap crop to prevent the spreading of PCN.

In the present study, we analyzed root exudates at one time point. For future experiments it could be interesting to also investigate the SolA concentration in root exudates collected at different timepoints and/or different plant phenological stages.

The hatching assay, using eggs of *G. pallida*, showed that SolA is extremely active in sub-nM concentrations, which is much lower than the values reported for glycoalkaloid hatching stimulants such as α-tomatine, which induces only average hatching rates at µM concentrations (Shimizu et al. 2020). Furthermore, from our results, *G. pallida* seems to be equally sensitive to SolA compared to *G. rostochiensis* and *G. tabacum*, which were studied by Sakata et al. (2020). However, the same study showed a maximum hatching rate of 20% by SolA in *G. pallida*, which is much lower than what we find here. Possibly, Sakata et al. (2020) used a different pathotype of *G. pallida*, which could explain this discrepancy.

Interestingly, the hatching rates induced by the root exudates from different species and genotypes quite well correlate with their SolA concentration and matches the hatching rates obtained with pure SolA, although a matrix effect seems to be present that increases hatching of SolA in root exudates. Indeed, for some root exudates, such as for *S. lycopersicum* cv. MicroTom, *S. pennelli* and *S. sisymbriifolium* cv. Quattro, the SolA levels are too low to explain their hatching rates of ~ 40–50%. The root exudates of these genotypes contain 1–5 pM SolA, whereas 40% hatching is only achieved by 50 pM SolA (Fig. 4b). Hence, the root exudate probably contains additional, unknown, hatching stimulants, causing a high hatching rate despite the low SolA concentration. As already discussed, tomatidine and α-tomatine may contribute to induce hatching, but are not very effective and alone cannot explain the hatching rates obtained (Shimizu et al. 2020). Other host species of PCN such as potato, are known to produce a range of hatching factors as well, like α-chaconine, α-solanine and solanidine (Stobiecki et al. 2003; Shakya and Navarre 2008), which induce hatching in *G. rostochiensis*, but only at nM concentrations (Ochola et al. 2020).

Moreover, root exudates may also contain hatching inhibitors that can interfere with the correlation between SolA and hatching rate. Finally, some of the other hatching stimulating compounds described in the literature were shown to induce hatching according to an optimum curve, and at very high concentrations act as inhibitors (Devine et al. 1996; Byrne et al. 1998). Further work should characterize the full spectrum of bioactive compounds in the root exudate that together determine the hatching of PCN.

In conclusion, we developed an efficient method for the analysis of SolA in root exudate and showed that there is substantial natural variation in its production by cultivated and wild tomato genotypes and the trap crop *S. sisymbriifolium*. Hatching assays revealed that there is a significant positive correlation between SolA concentration and hatching activity. The analytical method we have developed can potentially be used to support breeding for crop genotypes that induce less hatching and may therefore display reduced infection by PCN.

### *Author contribution statement*

Analytical chemical experiments were designed by KF, MLS, GK and AC and executed by KF, AC, YR van D and AG; growth, harvest and processing of plant samples was designed and executed by AG; hatching assays were designed and executed by LEV; study execution, data collection and preparation of the original draft was carried out by AG, KF, MLS, GK, and LEV. The corresponding authors, LD and HJB, participated in the research design, conceptualization, project administration, funding acquisition, project supervision, review and editing of the manuscript. All authors have read and agreed to the published version of the manuscript.

## Supplementary Information

Below is the link to the electronic supplementary material.

Supplementary file1 (DOCX 1810 kb)

Supplementary file2 (PNG 288 kb)

## Data Availability

The datasets generated during and/or analyzed during the current study are available from the corresponding author on reasonable request.

## Abbreviations

SolA: Solanoeclepin A
PCN: Potato cyst nematode
SPE: Solid phase extraction
