# Supplementary material for: UPLC-MS/MS analysis and biological activity of the potato cyst nematode hatching stimulant, solanoeclepin A, in the root exudate of Solanum spp
Source: Planta. 2021 Nov 2;254(6):112. doi: 10.1007/s00425-021-03766-2 (PMC8563560; doi:10.1007/s00425-021-03766-2)
Supplement: Supplementary file 1 — Supplementary file1 (DOCX 1810 kb) [file 425_2021_3766_MOESM1_ESM.docx]

**UPLC-MS/MS analysis and biological activity of the potato cyst nematode hatching stimulant, solanoeclepin A, in the root exudate of *Solanum* spp.**

Alessandra Guerrieri^1✝^, Kristýna Floková^1✝^, Lieke E. Vlaar^1✝^, Mario L. Schilder^1^, Gartjan Kramer^2^, Aleksandra Chojnacka^3^, Yannick R. van Dijk^1^, Harro J. Bouwmeester^1*^ and Lemeng Dong^1*^

**Supplementary files**

1 Plant Hormone Biology group,

2 Mass Spectrometry of Biomolecules

Swammerdam Institute for Life Sciences (SILS), University of Amsterdam, Amsterdam, The Netherlands

3 Biomolecular System Analytics

Van’t Hoff Institute for Molecular Sciences (HIMS), University of Amsterdam, Amsterdam, The Netherlands

^✝^these authors contributed equally

^*^Correspondence: [h.j.bouwmeester@uva.nl](mailto:h.j.bouwmeester@uva.nl) (H. J. B.), l.dong2@uva.nl (L.D.)

**Supplemental Table S1** The stability of SolA (2.5 pmol) in methanol, 0.2M formic acid in methanol and tomato root exudate (TRE) at 4°C over 2 hours, determined as a percentage recovery. The recovery is calculated as the mean peak area of SolA spiked before treatment to mean peak area of SolA spiked to dry sample matrix multiplied by 100 (1). Extraction recovery of the single-step SPE purification protocol. Experiments were performed using 5 ml of water (control) and aqueous extracts of TRE, spiked with 2.5 pmol SolA. Spiked extraction solvent (control) and TRE exudates were purified using polymer-based Oasis^®^ MAX columns (60 mg/3 cc, Waters) and analyzed by UPLC-MS/MS. Extraction recovery is calculated as the mean peak area of SolA spiked before purification to mean peak area of SolA spiked to purified sample matrix multiplied by 100 (2). All quoted values are means ± standard deviation (*n* =3^a^, 4^b^).

| **(1) Stability of SolA** | **methanol** | **0.2M formic acid/ methanol** | **TRE** |
| --- | --- | --- | --- |
| (%)^a^ | 84.9 ± 0.1 | 77.9 ± 1 | 85.9 ± 5 |
|  |  |  |  |
| **(2) Extraction recovery** | **control** | **TRE (5 ml)** |  |
| (%)^b^ | 93.9 ± 5 | 90.3 ± 2 |  |
|  | |  |  |

**Supplemental Table S2** The number of replicates, the name of the samples, the root mass expressed in grams of fresh weight (g FW) and the amount of SolA detected in the root exudates (RE) of different Solanaceous species. The amount of SolA is given as pmol per 300 ml of RE, pmol/ml of RE and pmol/g FW and pM.

| Replicate | Name | Root weight  (g FW) | pmol/pot  (300 ml RE) | pmol/ml RE | pmol/g FW | pM |
| --- | --- | --- | --- | --- | --- | --- |
| 1 | *S. lyc* Moneymaker | 4.62 | 73.850 | 0.246 | 15.985 | 246.168 |
| 2 | *S. lyc* Moneymaker | 2.55 | 66.182 | 0.221 | 25.954 | 220.608 |
| 3 | *S. lyc* Moneymaker | 2.3 | 26.720 | 0.089 | 11.618 | 89.068 |
| 4 | *S. lyc* Moneymaker | 2.88 | 41.536 | 0.138 | 14.422 | 138.452 |
| 5 | *S. lyc* Moneymaker | 3.29 | 93.677 | 0.312 | 28.473 | 312.256 |
| 1 | *S. lyc* MicroTom | 1.54 | 9.467 | 0.032 | 6.147 | 31.556 |
| 2 | *S. lyc* MicroTom | 1.2 | 14.842 | 0.049 | 12.368 | 49.472 |
| 3 | *S. lyc* MicroTom | 1.36 | 12.709 | 0.042 | 9.345 | 42.364 |
| 4 | *S. lyc* MicroTom | 1.66 | 27.380 | 0.091 | 16.494 | 91.268 |
| 5 | *S. lyc* MicroTom | 1.85 | 11.839 | 0.039 | 6.400 | 39.464 |
| 1 | *S. pimpinellifolium* | 7.09 | 125.491 | 0.418 | 17.700 | 418.304 |
| 2 | *S. pimpinellifolium* | 12.8 | 267.509 | 0.892 | 20.899 | 891.696 |
| 3 | *S. pimpinellifolium* | 8.45 | 198.023 | 0.660 | 23.435 | 660.076 |
| 4 | *S. pimpinellifolium* | 9.54 | 202.298 | 0.674 | 21.205 | 674.328 |
| 5 | *S. pimpinellifolium* | 8.18 | 106.926 | 0.356 | 13.072 | 356.42 |
| 1 | *S. habrochaites* PI127826 | 2.21 | 125.732 | 0.419 | 56.892 | 419.108 |
| 2 | *S. habrochaites* PI127826 | 2.68 | 67.720 | 0.226 | 25.269 | 225.732 |
| 3 | *S. habrochaites* PI127826 | 4.02 | 271.828 | 0.906 | 67.619 | 906.092 |
| 4 | *S. habrochaites* PI127826 | 4.74 | 158.285 | 0.528 | 33.393 | 527.616 |
| 5 | *S. habrochaites* PI127826 | 3.34 | 173.197 | 0.577 | 51.855 | 577.324 |
| 1 | *S. habrochaites* LA1777 | 4.1 | 66.176 | 0.221 | 16.141 | 220.588 |
| 2 | *S. habrochaites* LA1777 | 13.68 | 137.110 | 0.457 | 10.023 | 457.032 |
| 3 | *S. habrochaites* LA1777 | 7.1 | 42.574 | 0.142 | 5.996 | 141.912 |
| 4 | *S. habrochaites* LA1777 | 4.06 | 114.445 | 0.381 | 28.188 | 381.484 |
| 5 | *S. habrochaites* LA1777 | 6.46 | 78.010 | 0.260 | 12.076 | 260.032 |
| 1 | *S. sysimbriifolium* Pion | 10.58 | 159.608 | 0.532 | 15.086 | 532.028 |
| 2 | *S. sysimbriifolium* Pion | 5.42 | 8.479 | 0.028 | 1.564 | 28.264 |
| 3 | *S. sysimbriifolium* Pion | 3.84 | 0.000 | 0.000 | 0.000 | 0 |
| 4 | *S. sysimbriifolium* Pion | 4.29 | 52.182 | 0.174 | 12.164 | 173.94 |
| 5 | *S. sysimbriifolium* Pion | 3.67 | 42.893 | 0.143 | 11.687 | 142.976 |
| 1 | *S. sysimbriifolium* Quattro | 4.84 | 0.000 | 0.000 | 0.000 | 0 |
| 2 | *S. sysimbriifolium* Quattro | 6.29 | 0.000 | 0.000 | 0.000 | 0 |
| 3 | *S. sysimbriifolium* Quattro | 3.52 | 1.121 | 0.004 | 0.318 | 3.736 |
| 4 | *S. sysimbriifolium* Quattro | 1.96 | 0.000 | 0.000 | 0.000 | 0 |
| 5 | *S. sysimbriifolium* Quattro | 5.55 | 15.710 | 0.052 | 2.831 | 52.368 |
| 1 | *S. pennelli* | 2.2 | 18.358 | 0.061 | 8.344 | 61.192 |
| 2 | *S. pennelli* | 1.24 | 6.811 | 0.023 | 5.493 | 22.704 |
| 3 | *S. pennelli* | 2.35 | 3.764 | 0.013 | 1.602 | 12.548 |
| 4 | *S. pennelli* | 1.95 | 4.253 | 0.014 | 2.181 | 14.176 |
| 5 | *S. pennelli* | 2 | 40.830 | 0.136 | 20.415 | 136.1 |


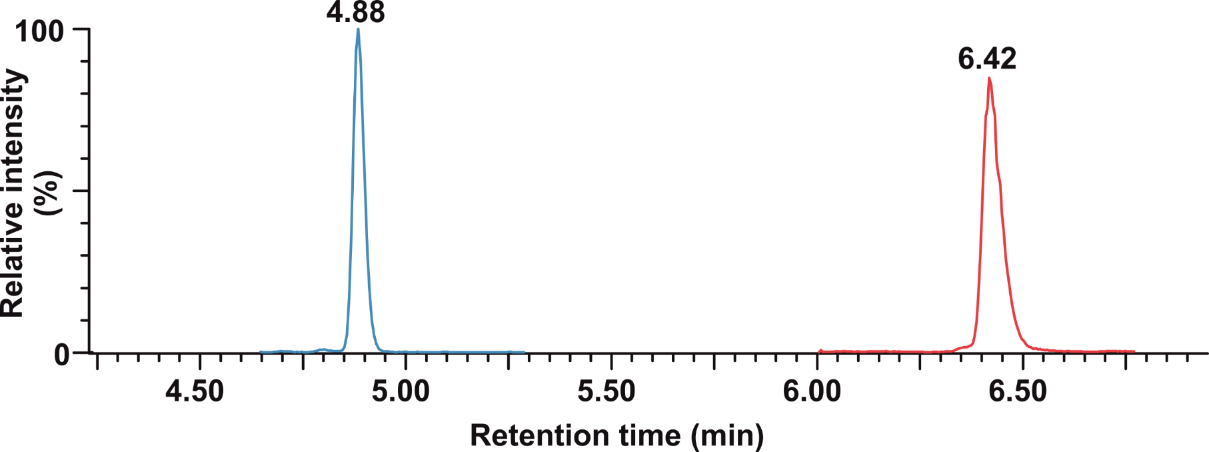


**Supplemental Fig. S1** The comparison of retention time and peak shape of SolA (0.5 pmol/injection) using separation on two different reversed-phase based UPLC columns - Acquity UPLC^®^ BEH C18 (blue line) and Acquity UPLC^®^ CSH C18 (red line, both 2.1 mm x 100 mm, 1.7 µm, Waters). MS data were recorded in the multiple-reaction monitoring (MRM) mode, using transition 499>399.


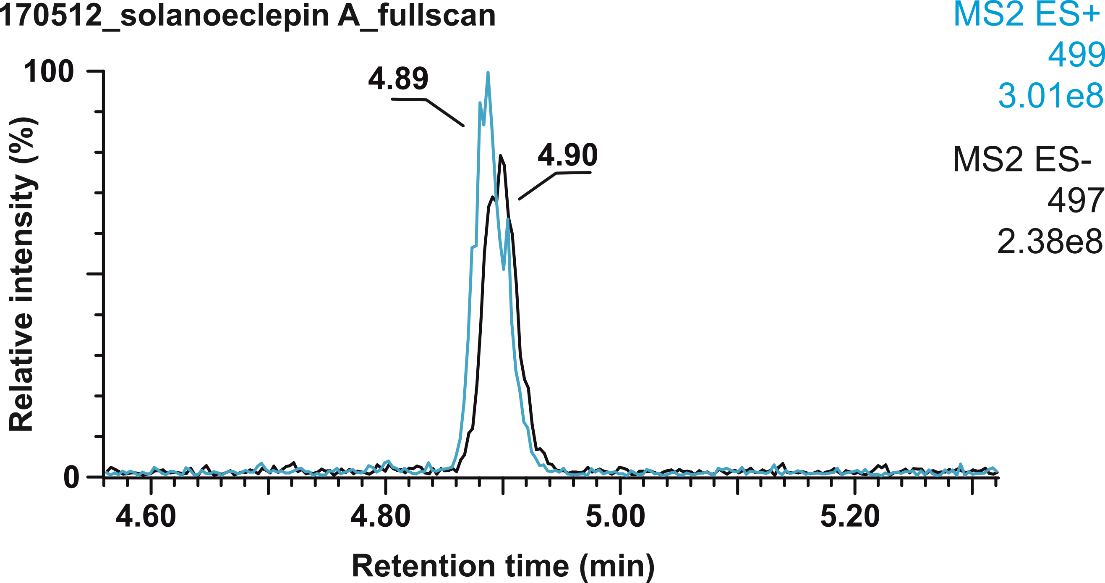


**Supplemental Fig. S2** The molecular ion intensity of SolA, obtained by UHPLC-MS with an electrospray interface (ESI), operating in positive (*m/z* = 499, blue line) and negative (*m/z* = 497, black line) ionization mode. The standard (5 pmol/5 µl) was injected onto an Acquity UPLC^®^ BEH C18 2.1 × 100 mm, 1.7µm column and separated by 9 min linear gradient using 15 mM additive formic acid in the mobile phase.


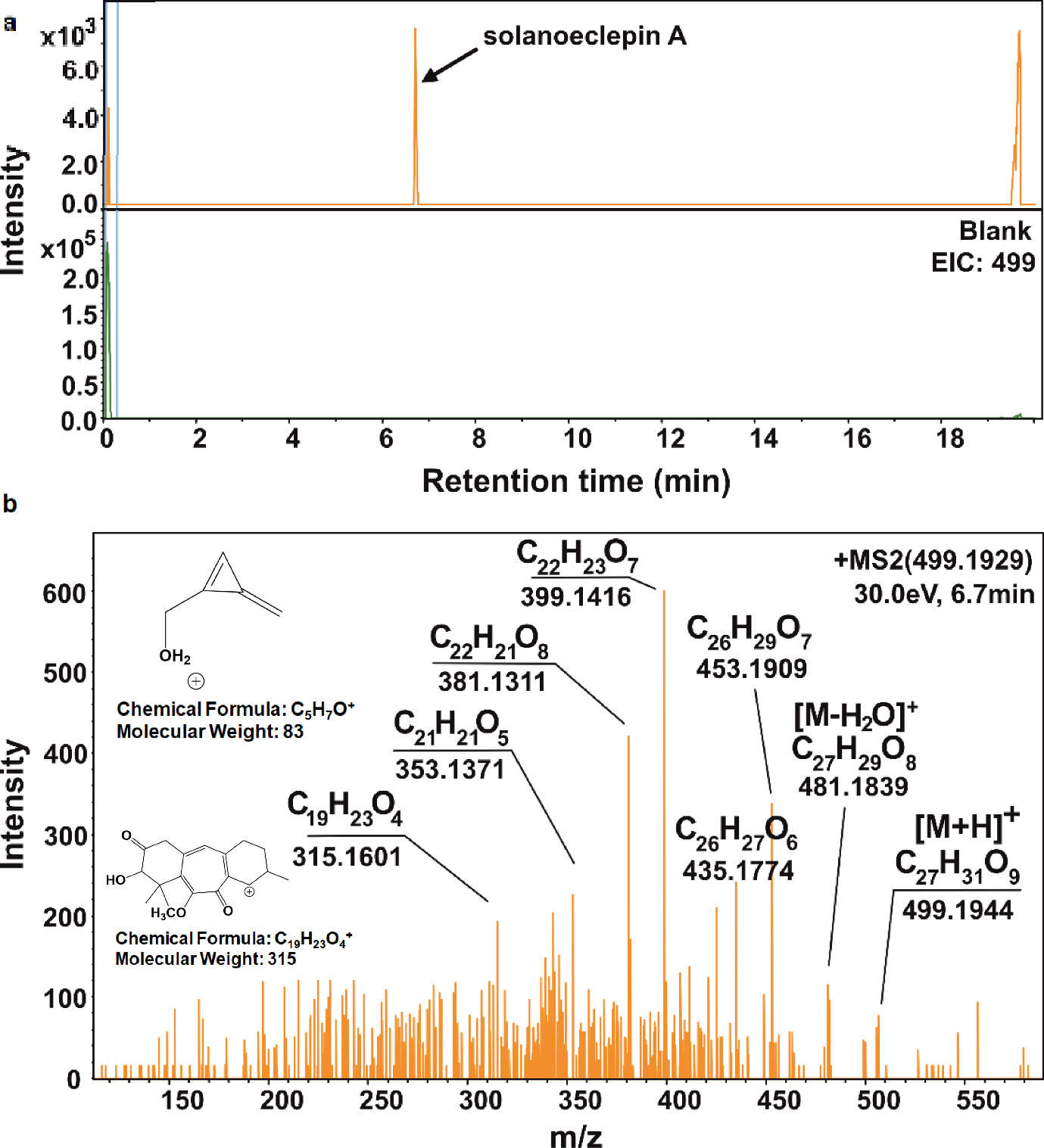


**Supplemental Fig. S3** LC-QTOF MS analysis of SolA standard. **a** LC-TIMS-TOF-MS product-ion spectra of SolA and structures of predicted fragmentation. The black arrow indicates the SolA peak. **b** MS2 mass spectrum of 499.1944 indicative of SolA [M+H]^+^.


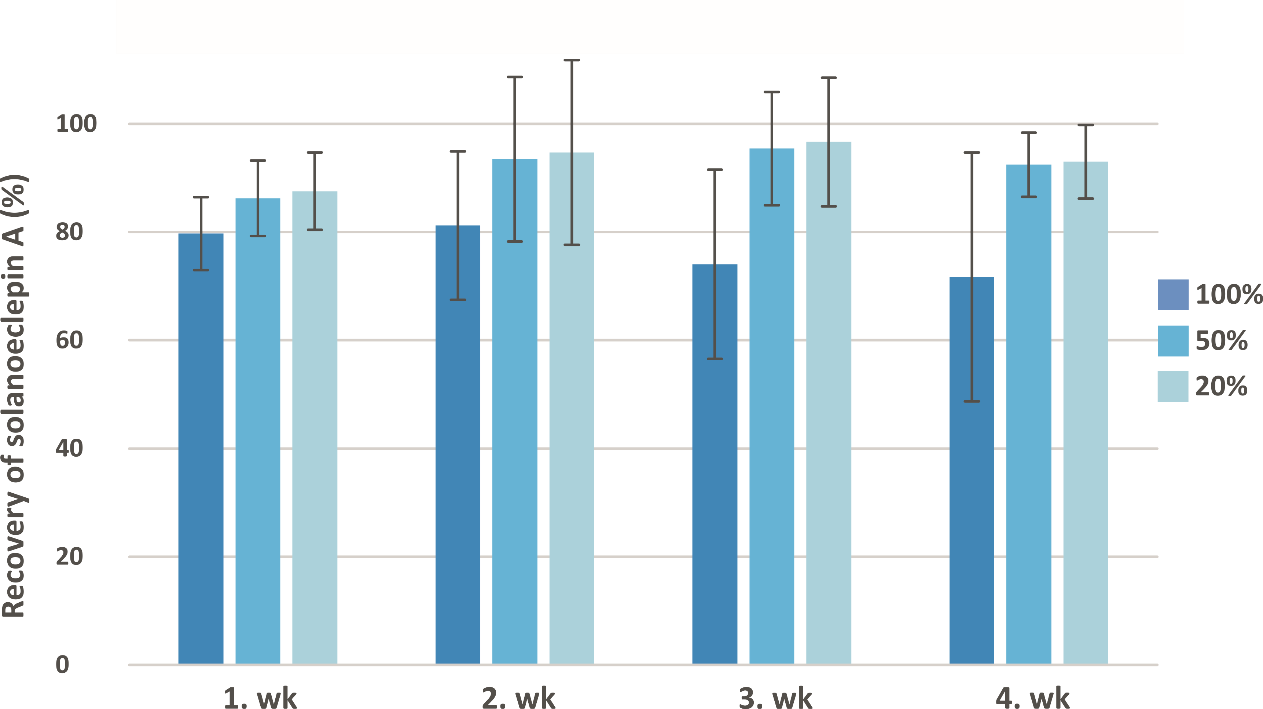


**Supplemental Fig. S4** The stability of SolA in 100% methanol and its 50% and 20% aqueous solutions over four weeks. To compensate for instrument response, the set of samples in duplicates were freshly prepared before each analysis as control. Following measurements contributed to the set of 1, 2, 3 or 4-week-old samples. Each bar represents the percentage of the mean peak area measured in the set of 1, 2, 3 or 4-week-old samples in octuplicates, compared to the average peak area of the standard, freshly prepared each week. Samples were dried in vacuo, reconstituted in the mobile phase and SolA content was analyzed by UHPLC-ESI(+)-MS/MS. Error bars represent the standard deviation of the mean (±SD, *n* =8).


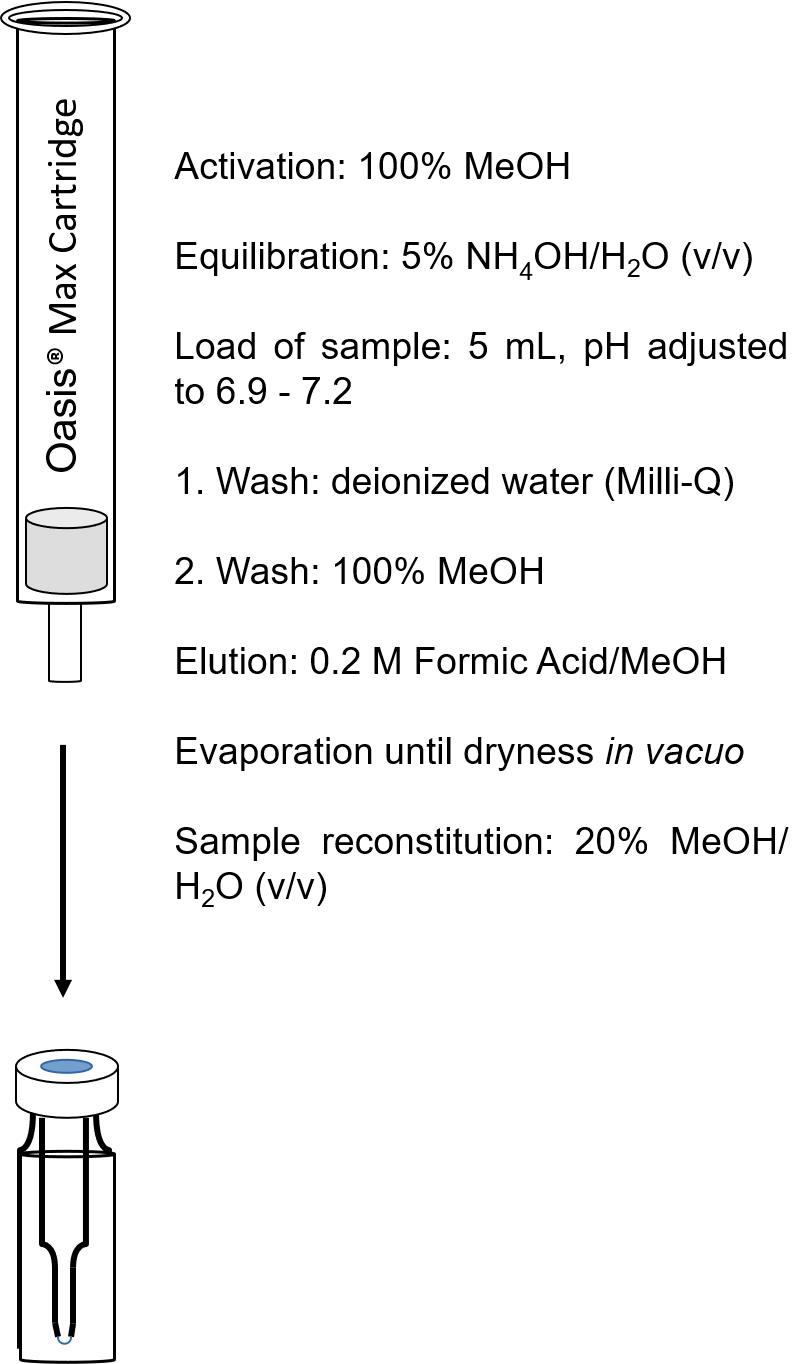


**Supplemental Fig. S5** Sample preparation procedure for SolA extraction using the polymer-based SPE Oasis® MAX column (Waters). 5 ml of sample was loaded onto the pre-conditioned cartridge and the SolA fraction was collected in 0.2 M FA/MeOH(v/v). Samples were evaporated until dryness, then reconstituted in 100 µl of 20% MeOH/H_2_O (v/v) for UHPLC-MS/MS analysis.


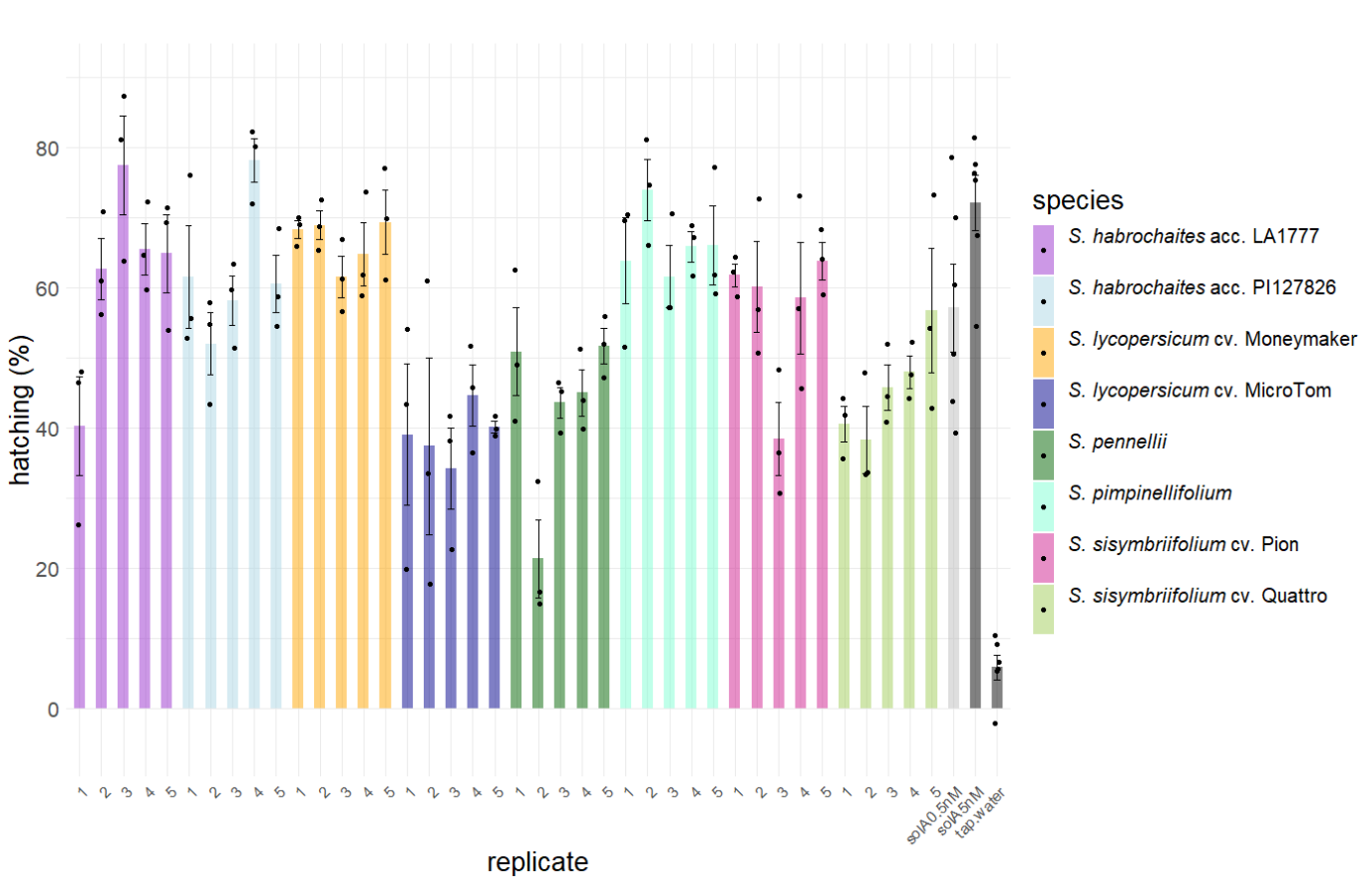


**Supplemental Fig. S6** Hatching percentages of *G. pallida* eggs treated with root exudates of solanaceaous species. Of each species, root exudate from 5 individual plants were harvested, and these were tested in triplicate on 50-100 *G. pallida* eggs. Error bar is standard error, *n* =3


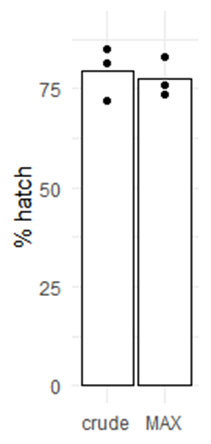


**Supplemental Fig. S7** Crude root exudate and partially MAX cartridge SPE purified root exudate were both analysed for their hatching activity towards *G. pallida* eggs.


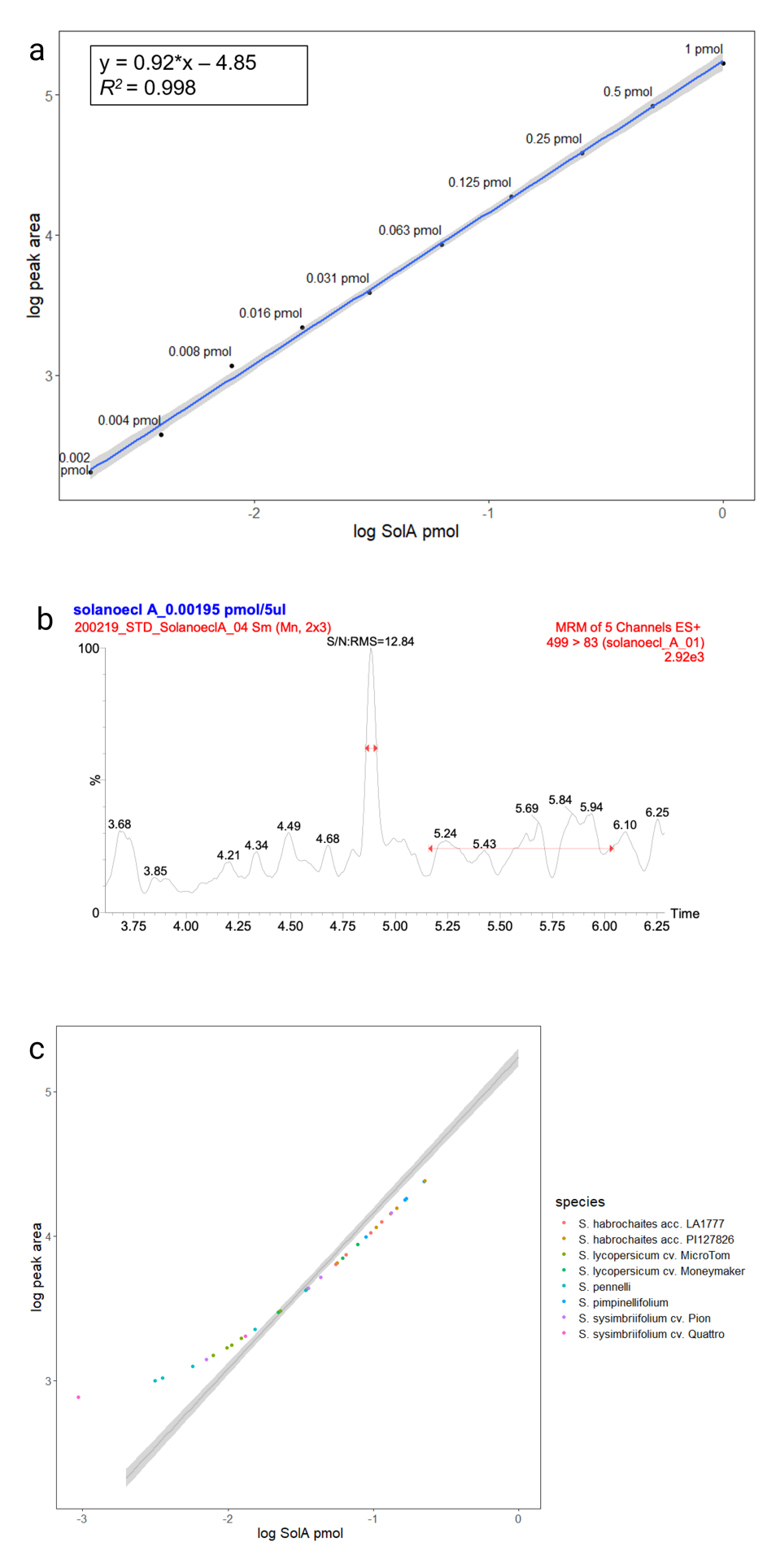


**Supplemental Fig. S8** Quantification of SolA. **a** Linear dynamic range of calibration curve of SolA across concentrations from 0.002 to 1 pmol per injection. *R^2^*= 0.998; coefficient of the slope b = 0.92, and intercept = -4.85. **b** Chromatogram of the diagnostic precursor-to-product ion transition at the limit of quantitation (LOQ: 0.002 pmol), signal to noise ratio = 12.84. **c** The standard curve (**a**) was used to calculate endogenous concentration levels of SolA in Solanaceae root exudates.


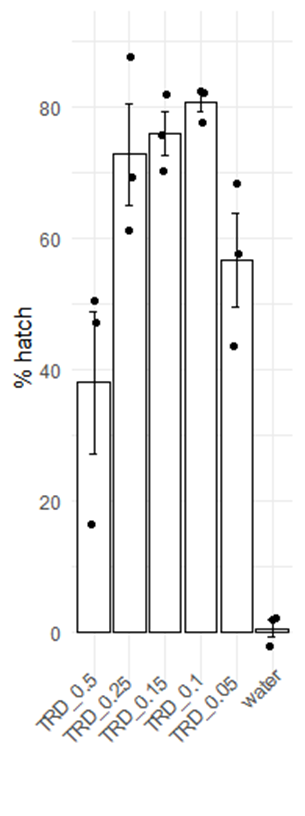


**Supplemental Fig. S9** Dilution series of root exudate (*S. lycopersicum* cv. Moneymaker) showing that there is an optimal dilution for highest hatching activity in *G. pallida*.


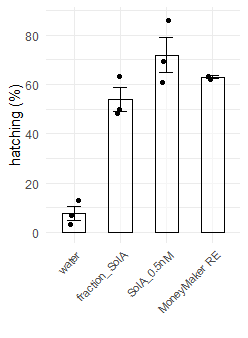


**Supplemental Fig. S10** Hatching of *G. pallida* in crude and fractionated root exudate of *S. lycopersicum* cv. Moneymaker. The root exudate was dissolved in 2% EtOH in water and tested for hatching of *G. pallida*. The SolA containing UHPLC fraction is compared with the crude exudate and a SolA standard of 0.5 nM.
