# Supplementary figures and images for: UPLC-MS/MS analysis and biological activity of the potato cyst nematode hatching stimulant, solanoeclepin A, in the root exudate of Solanum spp
Source: Planta. 2021 Nov 2;254(6):112. doi: 10.1007/s00425-021-03766-2 (PMC8563560; doi:10.1007/s00425-021-03766-2)

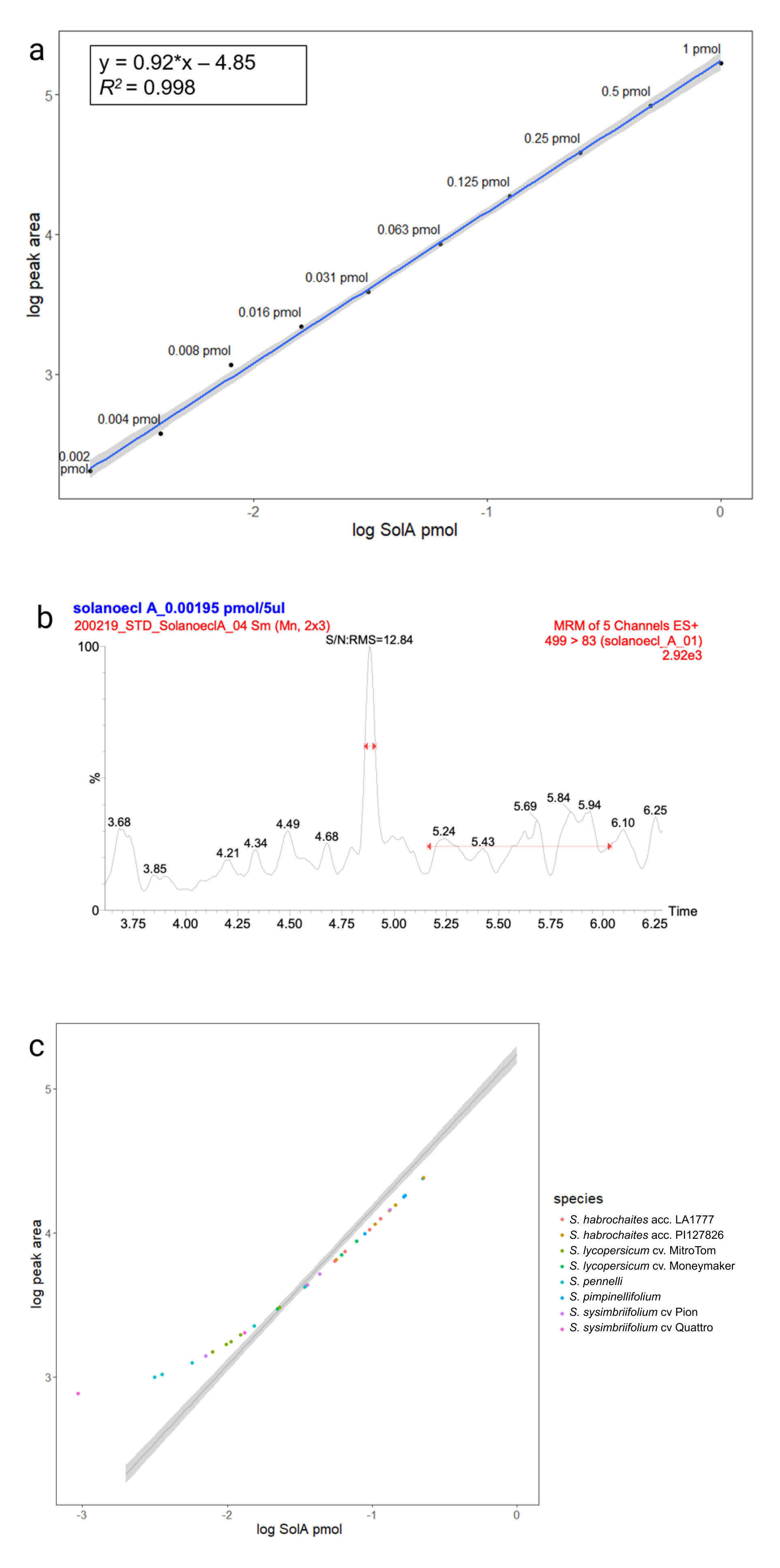

Supplement: Supplementary file 2 — Supplementary file2 (PNG 288 kb) [file 425_2021_3766_MOESM2_ESM.png]
